# Restless leg syndrome in a patient with celiac disease: a coincidence or an association?

**DOI:** 10.4103/0256-4947.51779

**Published:** 2009

**Authors:** Fady G. Haddad, Georges D. Maalouly, Joe I. Fahed, Mouin H. Jammal, Rita J. El Nemnoum

**Affiliations:** From the Department of Internal Medicine, Hotel-Dieu de France Hospital, Beirut, Lebanon

**To the Editor:** A myriad of extraintestinal manifestations of celiac disease have been described,^1^ but none have included restless leg syndrome (RLS). We report a case of celiac disease presenting with RLS. A 45-year-old woman was seen in June 2007 for exacerbation of sleep discomfort related to an uncomfortable sensation in the limbs at night that also resulted in daytime somnolence. The sensation decreased with movement and disappeared with walking. She also reported abdominal pain and diarrhea. Repeated colonoscopies in 2002 and 2006 were normal. Her medical history revealed a long-standing anemia refractory to oral iron supplementation and weight loss. A neurological exam was normal. Initial laboratory studies showed a hemoglobin of 9.8 g/dL (reference range, 13-15 g/dL) with a mean corpuscular volume of 82 fL, a mean corpuscular hemoglobin of 26.5 pg/cell (reference range, 3033 pg/cell) serum iron of 11.3 μmol/L (normal range, 8.8-32.4 μmol/L) and serum ferritin of 6.2 ng/mL (normal range, 30-400 ng/mL). Thyroid stimulating hormone, folic acid and vitamin B12 levels were normal. Her 25-hydroxyvitamin D was 7.2 ng/mL (normal, >20 ng/mL). Bone mass densitometry revealed osteoporosis at the spinal level (T score, −3.2). IgA endomysial antibodies and IgA tissue transglutaminase antibodies (>200 RU/mL, normal <20 RU/mL), and IgA antigliadin antibodies (30 UI/mL, normal <5 UI/mL) were positive. Upper gastrointestinal endoscopy revealed an atrophic duodenal mucosa. Duodenal biopsy showed severe villous atrophy with mononuclear infiltrate and epithelial cell damage. A diagnosis of celiac disease with secondary RLS was made. A gluten-free diet and intravenous iron supplementation abated the symptoms of RLS in 6 weeks. At the 6-month visit she had gained 8 kilograms and her serum ferritin level was 57 ng/mL. The patient was diagnosed as having secondary RLS and received parenteral iron supplementation for 5 days.

RLS occurs in 3% to 10% of the general population.^1^ It can be associated with a variety of underlying medical disorders, especially iron deficiency.^2^ Some authors have suggested that serum ferritin levels were inversely related to RLS severity, but even when serum concentration of ferritin were normal, ferritin was frequently reduced in the cerebrospinal fluid.^2^ RLS may be the only clinical manifestation of iron deficiency and when serum ferritin concentration is below 50 ng/mL, a cause of iron deficiency should be pursued.^3^ Asymptomatic celiac disease was the cause of iron deficiency in 10% of patients referred to a gastroenterologist^4^ and in 8.5% of patients with iron deficiency anemia unresponsive to oral iron therapy.^5^ Central nervous system manifestations occur in 10% of celiac disease patients.^6^ Cerebellar ataxia is the most frequent, but myoclonus, internuclear opthalmoplegia, dementia and multifocal leucoencephalopathy have all been reported.^6^ Peripheral neuropathy has also been reported in association with celiac disease. It occurs generally late in the course of the disease and occasionally during exacerbations of steatorrhea.^7^ Recent studies suggest that the mechanism is immunological rather than related to vitamin B deficiency.^5^

To our knowledge, RLS has never been reported as a neurological manifestation of celiac disease. The association of these two diseases may be underestimated. Our patient presented with a simultaneous exacerbation of RLS, abdominal pain and diarrhea. Substantial improvement of her symptoms was achieved by iron supplementation and a gluten-free diet. Iron deficiency or an unknown immune mechanism could explain the association of these two diseases.
